# Primary health care approach to rheumatic heart disease management

**DOI:** 10.4102/safp.v68i1.6255

**Published:** 2026-03-31

**Authors:** Ruchika Meel, Ramprakash Kaswa

**Affiliations:** 1 Department of Internal Medicine, Faculty of Health Sciences, University of the Witwatersrand, Johannesburg, South Africa; 2 Department of Family Medicine and Rural Health, Walter Sisulu University, Mthatha, South Africa; 3 Department of Health, Mthatha Regional Hospital, Mthatha, South Africa

**Keywords:** rheumatic heart disease, primary health care, acute rheumatic fever, Group A streptococcus, prophylaxis

## Abstract

The primary health care (PHC) approach to rheumatic heart disease emphasises preventing initial episodes of rheumatic fever through primary prevention, avoiding recurrences by secondary prevention and ensuring long-term follow-up and an effective referral system. Primary care providers act as gatekeepers of community healthcare needs. They are heavily involved in community engagement, primary prevention, continuity of care and are part of strong PHC referral networks. This positions them ideally to participate in the prevention and early detection of rheumatic heart disease. The main aim of this CPD article is to guide the early identification and management of rheumatic heart disease within PHC settings.

## Introduction

Rheumatic heart disease (RHD) is a significant and increasingly prominent public health issue worldwide, affecting around 50 million people globally. The prevalence of RHD has increased 1.7 times since 1990.^1^ The overall increase in cases is likely because of factors such as population growth, improved awareness, expanded access to diagnostics, better survival rates in certain contexts and the persistent nature of the disease itself.^2^

The burden of RHD varies around the world. It has notably decreased in high-income countries because of improved sanitation, better housing, higher living standards and increased access to healthcare and antibiotics. In contrast, RHD mainly persists among rural, poor and marginalised communities with limited or no access to primary health care (PHC). The disease remains common in areas marked by poverty, overcrowding and healthcare inequalities.^2,3^ The African continent bears a significant share of the global RHD burden, with 32.9 million active cases and more than 319 000 new cases annually. Sub-Saharan Africa (SSA) accounts for 23% of global RHD cases, with an estimated prevalence of about 10 cases per 1000 people.^3,4^

Rheumatic heart disease remains the most common cause of acquired heart conditions among young adults in low- and middle-income countries (LMICs). Rheumatic heart disease accounts for an estimated 10.67 million disability-adjusted life years (DALYs) and causes around 360 000 deaths annually. This makes it the ninth leading cause of global mortality from invasive Group A Streptococcus (GAS) infections.^4^ The 2-year case fatality rate observed in the Global Rheumatic Heart Disease Registry was 16.9%, with notably higher mortality rates in low-income countries.^5^

Rheumatic heart disease often causes serious complications, including infective endocarditis, heart failure, arrhythmias, pulmonary hypertension, systemic embolic events and stroke.^2,6^ It is the third most common cause of heart failure in adults in SSA. In some regions of Africa, there is a changing pattern of the disease, characterised by a decline in cases of acute rheumatic fever (ARF) and RHD in children, while a high rate of congestive heart failure caused by RHD remains among adults. This indicates a shift in ARF from a mainly childhood disease to a more endemic condition of RHD in adults.^6,7^

The impact of RHD has gained worldwide recognition, with the World Health Assembly (WHA) passing a historic resolution recognising it as a major global health concern. This resolution urges Member States to take vital actions, including strengthening multisectoral efforts to identify and prevent RHD at the PHC level.^1^ The main aim of this CPD article is to assist primary care providers with the effective screening and early detection of RHD. It also highlights the importance of active case finding and the prevention of ARF through both primary and secondary measures within the PHC system.

## Natural history and progression

**FIGURE 1:**
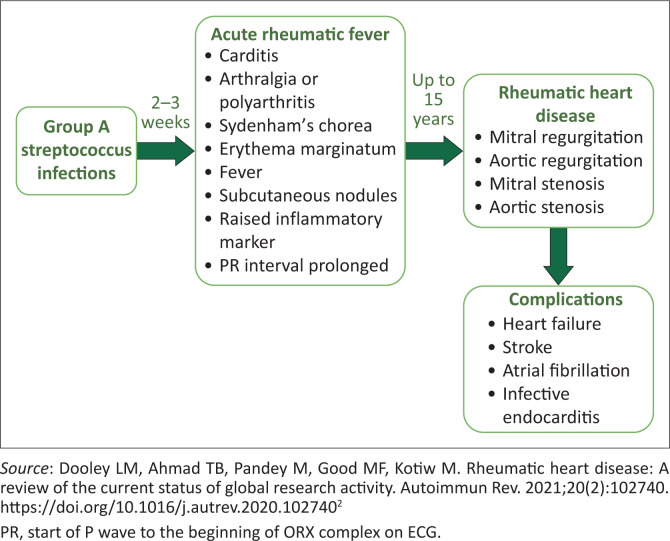
Natural history of acute rheumatic fever.

Acute rheumatic fever is an autoimmune inflammatory reaction caused by GAS infection, and repeated episodes can lead to irreversible heart damage, as shown in Figure 1. Acute rheumatic fever usually presents 2–3 weeks after a GAS throat infection or, in some cases, skin infections.^2,8^ Untreated GAS increases the risk of recurrent ARF and results in cumulative valvular damage. Repeated ARF episodes can worsen existing RHD and accelerate valvular deterioration.^3^ The autoimmune response triggered by GAS can affect multiple organs:

- **Carditis:** This is the most serious and critical complication of ARF, as it can cause permanent damage to at least one of the four heart valves, leading to RHD. Myocarditis, pericarditis and heart block are other features.^1^
- **Arthritis:** Arthralgia or polyarthritis, typically affecting large joints (wrists, elbows, knees and ankles), and often migratory and initially asymmetrical.^2^
- **Chorea (Sydenham’s chorea):** It is a delayed sign of ARF, identified by jerky, uncontrollable body movements. It is more common in women and appears late in the progression of ARF.^2^
- **Erythema marginatum:** A rash characterised by a pale centre, usually appearing on the back, and occurring early with concurrent carditis.
- **Subcutaneous nodules:** They are generally under two cm, movable, painless and found on the extensor surfaces of the arms, legs and head.^2^

Many patients with ARF may experience subclinical or unnoticed initial events that lead to recurrent insults. The disease often remains undetected for years, with over 85% diagnosed only when significant heart damage occurs.^9^ The mitral valve is most commonly affected, typically with mitral regurgitation (MR), followed by the aortic valve, which may experience regurgitation or stenosis. Multiple valve involvement, including tricuspid regurgitation (TR), is associated with lower survival rates.^6^

## Factors contributing to rheumatic heart disease

### Medical factors

Recurrent and untreated streptococcal throat infections are the primary cause of rheumatic fever. Repeated episodes of rheumatic fever result in cumulative damage to the heart valves, especially the mitral and aortic valves.^8^

### Genetic susceptibility

Some individuals present a stronger autoimmune response to streptococcal antigens. Their immune system attacks cardiac tissue after reacting to streptococcal proteins.^2^

### Socio-environmental factors

Poor living conditions and overcrowding increase the risk of streptococcal infection transmission. Lack of sanitation and hygiene elevates the chance of recurrent infections.^8^

### Health system factors

Limited access to PHC, delayed diagnosis and absence of primary and secondary prophylaxis for GAS, including long-term penicillin prophylaxis after rheumatic fever. Lack of awareness among the public and PHC providers lead to under-diagnosis and inadequate follow-up. There is also a lack of prophylaxis to prevent the recurrence of rheumatic fever.

### Demographic factors

Children and adolescents are more vulnerable to ARF after initial GAS infection. Females may face a slightly higher risk, often because of interactions of the immune system. Some ethnic groups or indigenous populations, where GAS infections are endemic or healthcare access, is limited.^3,10^

## Diagnosis of rheumatic heart disease

### Jones criteria

The Jones criteria are used to diagnose ARF and require evidence of a recent GAS infection, along with either two major manifestations or one major and two minor manifestations, as shown in Table 1. It begins with an initial step to determine whether a patient is from a low-risk or moderate- to high-risk population. This risk stratification aims to improve diagnostic accuracy in high-risk or endemic areas:^1,11^

- Low-risk populations: those with an incidence of ARF in children (5–14 years) < 2 per 100 000 per year or an RHD prevalence of ≤ 1 per 1000 population per year.^3^
- Moderate- or high-risk populations: those that have higher incidence and prevalence than the low-risk criteria.^3^

**TABLE 1:** Jones criteria for acute rheumatic fever.

Low-risk population	High-risk population
**Major criteria**	
Carditis (clinical or subclinical)	Carditis (clinical or subclinical)
Arthritis - only polyarthritis	Arthritis - monoarthritis or polyarthritis
Chorea	Polyarthralgia
Erythema marginatum	Chorea
Subcutaneous nodules	Erythema marginatum
	Subcutaneous nodules
**Minor criteria**	
Polyarthralgia	Monoarthralgia
Hyperpyrexia (≥ 38.5 °C)	Hyperpyrexia (≥ 38.5 °C)
ESR ≥ 60 mm/h and/or CRP ≥ 3.0 mg/dl	ESR ≥ 60 mm/h and/or CRP ≥ 3.0 mg/dl
Prolonged PR interval	Prolonged PR interval

*Source*: WHO guideline on the prevention and diagnosis of rheumatic fever and rheumatic heart disease. Geneva: World Health Organization; 2024^12^

ESR, erythrocyte sedimentation rate; PR interval, start of the P wave to the beginning of the QRS complex interval.

**TABLE 2:** Screening criteria for rheumatic heart disease.†

Valvular pathology in rheumatic heart disease
**Mitral Regurgitation (requires all the following)**
In individuals weighing < 30 kg: MR jet length ≥ 1.5 cm
In individuals weighing ≥ 30 kg: MR jet length ≥ 2.0 cm
MR jet is observed in at least one view
MR jet is observed in at least two consecutive frames
**Aortic regurgitation (requires all the following)**
Any Aortic regurgitation
Observed in at least one view
Observed in at least two consecutive frames
**Mitral stenosis**
Restricted leaflet motion with reduced valve opening

*Source*: Rwebembera J, Marangou J, Mwita JC, et al. 2023. World Heart Federation guidelines for the echocardiographic diagnosis of rheumatic heart disease. Nat Rev Cardiol. 2024;21(4):250–263. https://doi.org/10.1038/s41569-023-00940-9^3^

MR, mitral regurgitation.

† , Video link for parasternal long-axis view of mitral valve thickening and calcification: https://ars.els-cdn.com/content/image/1-s2.0-S2468644122001153-mmc1.mp4; Video link for parasternal short axis view of mitral valve thickeningpathology: https://ars.els-cdn.com/content/image/1-s2.0-S2468644122001153-mmc2.mp4.

Despite being the standard criteria, PHC providers often find the Jones criteria difficult to apply, which can lead to under-diagnosis of ARF,^1,4^ especially in the absence of specific clinical signs in the early stages of the disease and high false-negative throat culture rate, particularly weeks after infection in regions with extensive antibiotic use. Limited access to laboratory testing and the subjective nature of certain symptoms make consistent application challenging to PHC providers.

### Laboratory-based tools

Throat culture remains the gold standard for diagnosing pharyngitis caused by GAS, a precursor to ARF and RHD.^5^ The following tests can identify prior GAS infection:

- Serological tests to identify immune responses to bacterial enzymes (e.g. anti-streptolysin O, anti-DNase B, anti-hyaluronidase, anti-NADase and anti-streptokinase).
- Molecular point-of-care (POC) testing.
- Nucleic acid detection: real-time polymerase chain reaction (PCR).

### Echocardiography

Echocardiography is the most sensitive and gold standard method for diagnosing RHD owing to the following features:^1,13^

- Direct visualisation of valvular pathology.
- Detection of subclinical carditis.
- Integration of morphological and functional abnormalities.
- Assessment of disease severity and physiological effects.
- Identification of alternative diagnoses.

The WHO also recommends using point-of-care ultrasound (POCUS) to early identify rheumatic fever carditis and RHD in children, adolescents and adults in PHC settings where standard echocardiography is unavailable.^6^

## Prevention

**FIGURE 2:**
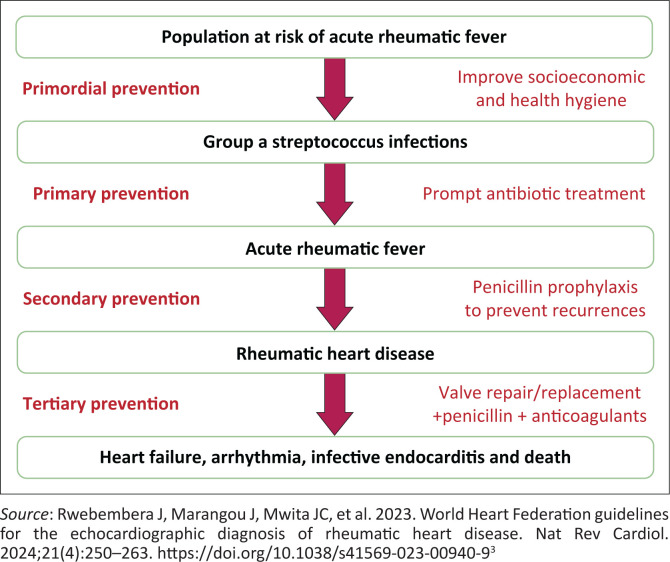
Stages of prevention of rheumatic heart disease.

Prevention of RHD is conceptualised across four levels of care (see Figure 2):

- Primordial prevention: aims to decrease GAS infections by enhancing living conditions and sanitation, as well as promoting community awareness and encouraging health-seeking behaviour. This serves as the fundamental strategy for eliminating RHD.^2^
- Primary prevention: involves treating GAS infections to prevent ARF and disease progression. In PHC settings where POC testing for GAS pharyngitis is unavailable, a clinical rule for suspected cases is recommended as a cost-effective approach to diagnosis.^2,3^
- Secondary prevention: seeks to prevent the recurrence of ARF through antibiotic prophylaxis to stop RHD development and progression. This is regarded as a fundamental part of RHD management.^3,5^
- Tertiary prevention: focuses on treating and managing RHD complications with medications for symptomatic relief and cardiac interventions, including surgery.^9^

## Screening criteria for active case finding of rheumatic heart disease

Active case finding for RHD requires echocardiography to identify valvular changes in individuals without a history of ARF or RHD. This method can detect a variety of valvular alterations associated with different risks of disease progression. The screening criteria are designed to identify potential cases in PHC settings where RHD is common, and resources such as equipment and healthcare personnel are limited. They are intended for individuals aged 20 years or younger. Based on the best available evidence, these criteria aim to maximise sensitivity as a screening tool, even though this may lower specificity.^1,14^

## Management of RHD

Management strategies for RHD encompass a multifaceted approach that includes eradication and prevention of GAS infection, symptomatic medical therapy, interventional procedures and surgery.

### Secondary antibiotic prophylaxis

Long-acting penicillin remains the primary preventive measure. Benzathine penicillin G (BPG) every 3–4 weeks to prevent recurrent ARF episodes.^14^ The South African national RHD prevention guideline recommends BPG doses of 600 000–900 000 units for individuals weighing less than 30 kg and 1,200 000 units for those weighing 30 kg or more.^15^ The oral alternative is phenoxymethyl penicillin. Oral cephalosporins and macrolides are alternative secondary prophylactic agents for penicillin-allergic patients. The prophylaxis duration is 5 years after the last attack or until the patient reaches 18 years of age, provided there are no signs of carditis. It extends to 10 years or until 25 years of age in individuals with established cardiac disease and is lifelong for those with severe cardiac disease or post-valve replacement.^1,15^

### Medical management of established rheumatic heart disease

Patients with established, symptomatic moderate to severe valvular disease require pharmacological treatment primarily to relieve symptoms rather than to change outcomes.

#### Heart failure

Use loop diuretics and spironolactone and reduce afterload with vasodilator therapy (e.g. angiotensin-converting enzyme inhibitors and angiotensin II receptor blockers). Digoxin and beta-blockers can also be considered based on individual patient needs.^1,5^

#### Atrial fibrillation

Oral anticoagulation (warfarin) is essential for atrial fibrillation (AF) management and for patients with prosthetic valves. Novel oral anticoagulants (e.g. rivaroxaban, dabigatran) may be considered based on individual patient indications.^3,5^

### Interventional and surgical treatment

Percutaneous balloon valvuloplasty for patients experiencing symptoms; alternative options include cardiac surgery (valve repair or replacement).^7,8^

### Preventive care

Including good dental hygiene, vaccinations and pregnancy planning. Pregnant women with RHD require a multidisciplinary team comprising cardiologists, obstetricians and paediatricians.^9,10^

### Challenges in the management of RHD

Here are current challenges of RDH management in PHC:

- Lack of awareness and low health literacy regarding RHD reported in a qualitative study from nine African countries.^11^
- Under diagnosis and lack of medical training among primary care providers.^13^
- Delayed diagnoses and treatment initiations in PHC settings.^11^
- Poor adherence to primary and secondary prophylaxis.^14^
- Scarcity of skilled health professionals.

### Opportunities in the management of RHD

There are opportunities in PHC for the management of RHD, which include:

- Implementation of the echocardiographic screening tool and POCUS training within the PHC setting to identify children and adolescents at risk, particularly in endemic regions. Point-of-care ultrasound training should be integrated into the Bachelor of Medicine and Bachelor of Surgery (MBChB) curriculum and into additional training for the existing primary care workforce.
- Early detection and prompt treatment of streptococcal pharyngitis with penicillin or appropriate antibiotics.
- Integrated RHD follow-up care at primary care clinics ensures patients stick to their management plans.^15^
- Standardised protocols in primary care clinics for managing sore throats, rheumatic fever and suspected RHD.
- Consolidate referral systems from primary care to specialised advanced care.
- Community awareness campaigns on the importance of treating sore throats in school health programmes to identify children at risk.
- Rehabilitation and supportive care for patients with established RHD who are not candidates for surgical interventions.
- Integrating RHD programmes into wider PHC and NCD frameworks with comprehensive community-oriented primary care by multidisciplinary teams.

## Conclusion

Rheumatic heart disease remains a neglected and preventable cardiovascular disease within unequal healthcare systems. Addressing RHD requires a multisectoral approach. Primary health care serves as the community’s first point of contact and is vital for RHD prevention through primary and secondary prophylaxis and early detection through ongoing screening. Managing RHD involves community education, easy access to care and appropriate referrals. The PHC approach has the potential to reduce the RHD burden, prevent complications and improve long-term outcomes. Primary health care is crucial to breaking this cycle, as RHD often results from untreated or recurrent Group A streptococcal infections.

## References

[1] Chinawa JM, Chinawa AT, Ogbuka FN, Elobuike PC, Nwankwo O. The prevalence rates of acute rheumatic fever in Africa: A systematic review and meta-analysis. Sage Open Pediatr. 2025;12:30502225251357145. 10.1177/30502225251357145 40735677 PMC12304603

[2] Dooley LM, Ahmad TB, Pandey M, Good MF, Kotiw M. Rheumatic heart disease: A review of the current status of global research activity. Autoimmun Rev. 2021;20(2):102740. 10.1016/j.autrev.2020.102740 33333234

[3] Rwebembera J, Marangou J, Mwita JC, et al. 2023. World Heart Federation guidelines for the echocardiographic diagnosis of rheumatic heart disease. Nat Rev Cardiol. 2024;21(4):250–263. 10.1038/s41569-023-00940-9 37914787

[4] Tibbutt DA. Valvular heart disease is changing – A challenge for Africa. South Sudan Med J. 2015;8(4):86–89.

[5] Peters F, Karthikeyan G, Abrams J, Muhwava L, Zühlke L. Rheumatic heart disease: Current status of diagnosis and therapy. Cardiovasc Diagn Ther. 2020;10(2):305–315. 10.21037/cdt.2019.10.07 32420113 PMC7225445

[6] World Health Organization. Rheumatic heart disease key facts [homepage on the Internet]. 2020 [cited 2025 Sept 15]; p. 1–4. Available from: https://www.who.int/news-room/fact-sheets/detail/rheumatic-heart-disease

[7] Meel R. Valvular heart disease and its growing impact in South Africa. SA Hear. 2024;21(3):204–207. 10.24170/21-3-6890

[8] Kumar RK, Antunes MJ, Beaton A, et al. Contemporary diagnosis and management of rheumatic heart disease: Implications for closing the gap: A scientific statement from the American Heart Association. Circulation. 2020;142(20):E337–E357. 10.1161/CIR.0000000000000921 33073615

[9] Zühlke L, Karthikeyan G, Engel ME, et al. Clinical outcomes in 3343 children and adults with rheumatic heart disease from 14 low- and middle-income countries. Circulation. 2016;134(19):1456–1466. 10.1161/CIRCULATIONAHA.116.024769 27702773

[10] Messika-Zeitoun D, Baumgartner H, Burwash IG, et al. Unmet needs in valvular heart disease. Eur Heart J. 2023;44(21):1862–1873. 10.1093/eurheartj/ehad121 36924203

[11] Moloi H, Zühlke L, Engel M, Daniels K. Health system factors influencing rheumatic heart disease prevention in nine African countries. J Public Health Africa. 2025;16(1):1–17. 10.4102/jphia.v16i1.1485

[12] WHO guideline on the prevention and diagnosis of rheumatic fever and rheumatic heart disease. Geneva: World Health Organization; 2024. 39631006

[13] Murugasen S, Abdullahi LH, Moloi H, et al. Burden of disease and barriers to comprehensive care for rheumatic heart disease in South Africa: An updated systematic review protocol. BMJ Open. 2023;13(6):e073300. 10.1136/bmjopen-2023-073300 PMC10254900 37263687

[14] Aliyu IA, Bala JA, Yusuf I, et al. Rheumatic heart disease burden in Africa and the need to build robust infrastructure. JACC Adv. 2024;3(12):101347. 10.1016/j.jacadv.2024.101347 39817077 PMC11734022

[15] Cornick R, Picken S, Wattrus C, et al. The Practical Approach to Care Kit (PACK) guide: Developing a clinical decision support tool to simplify, standardise and strengthen primary healthcare delivery. BMJ Glob Health. 2018;3(suppl 5):e000962. 10.1136/bmjgh-2018-000962 PMC6195147 30364419

